# Insects as source of phenolic and antioxidant entomochemicals in the food industry

**DOI:** 10.3389/fnut.2023.1133342

**Published:** 2023-03-01

**Authors:** Jorge A. Torres-Castillo, Fabián E. Olazarán-Santibáñez

**Affiliations:** ^1^Instituto de Ecología Aplicada, Universidad Autónoma de Tamaulipas, Ciudad Victoria, Tamaulipas, Mexico; ^2^Unidad Académica Multidisciplinaria Mante, Universidad Autónoma de Tamaulipas, Ciudad Mante, Tamaulipas, Mexico; ^3^Facultad de Medicina Veterinaria y Zootecnia, Universidad Autónoma de Tamaulipas, Ciudad Victoria, Tamaulipas, Mexico

**Keywords:** antioxidants, bioactive compounds, edible insects, entomophagy, nutraceuticals

## Abstract

Edible insects are a natural resource with profound interest in the food industry. Not only because of their nutritional content and technical production advantage, but also for the presence of bioactive compounds known as entomochemicals. These include phenolic, alkaloid, and terpenoid compounds, as well as amino acids derivatives, among others. This work is focused on phenolic compounds, which have been the best characterized due to their role in food development and bioactive properties. The major taxonomic orders studied in this regard include Orthoptera, Coleoptera, and Lepidoptera, whose edible specimens have antioxidant effects provided by the phenolic compounds contained therein. The use of these insects in the development of nutritious foods will enhance the number of options available for the human population. However, depth research is still needed to guarantee the aforementioned bioactivity in processed foods and ensure its innocuity, thus minimizing the risk of allergic reactions and allowing the full utilization of edible insect species in the food industry. Phenolic derived from edible insects portray an opportunity to improve high quality food, as an alternative to diversify and complement an adequate and functional diet. Future development foods supplemented with insects must consider the preservation of potential benefits of not only nutrients, also de nutraceuticals.

## 1. Introduction

Insects are important components of the ecosystem, where they perform essential tasks for its proper function and maintenance. These insects can be pollinizers, phytophagous, predators, parasites, parasitoids, detritivores, among others (1). Over 2,000 arthropod species, including insects, have been linked with entomophagy (enthomos, insect; phagous, feeding) throughout the historical interaction between humans and their environment. Most of edible insects are mainly included in Coleoptera, Lepidoptera, Orthoptera, Diptera, Hymenoptera, Hemiptera, and Isoptera. Some of the most consumed insect species are typically considered as pests; therefore, their consumption suggests this activity as a form of population control which, in turn, allows the integral management of a natural source of nutrients for the growing human population (2–6).

Currently, most insect pests affecting crops are destroyed with insecticides, which requires a large monetary investment to destroy a natural resource that could be otherwise used in the food industry; further, this also results in environmental pollution, biodiversity losses, and health problems caused by the use of agrochemicals (7). Therefore, wider and improved knowledge, technological development, and management strategies could provide pest insect species with the same potential as those reared massively (i.e., biological control) so they can be exploited in the food or medical industries.

The ingestion of insects has been practiced by diverse cultures throughout history, mostly as a source of nutrition, but also in cultural or religious practices. In addition, these insects could have been consumed raw or cooked, thus resulting in a wide variety of presentations (8, 9). This custom is now considered as an alternative means to satisfy the current and future food demand of the human population (10). However, the success of entomophagy is strongly tied with the acceptance of these insects as a common source of nourishment; especially in fully developed countries, where this practice is not widely accepted (11). An innovative preparation and presentation of insects as food, along with proper merchandising of entomophagy as a social trend, are essential for its success (10). Insects are glimpsed as an important source of animal protein because they have similar energetic content as chicken, beef, and pork; further, their content of fat, carbohydrates, fiber, and minerals is also nutritionally relevant (12). Moreover, the high scale production of edible insects is much more efficient, as they require less water and their CO_2_ footprint is minimal, making of them a truly sustainable food alternative (13, 14). Previous studies concerning the potential of insects as food address their nutritional content and, recently, most of them focused on the content of bioactive compounds (i.e., entomochemicals), especially in insect-specific secondary metabolites (15–18). The present study shows a compendium of the previous research exploring the potential of edible insects in the development of food, keeping a highlighted interest for entomochemicals of phenolic and antioxidant nature.

### 1.1. Diversity of entomochemicals

Within the wide variety of edible insects and the species classified as potentially edible, several types of entomochemicals have been reported as having bioactive properties with great similarity to those found in plants, animals, and microorganisms. Among these are found phenolic and alkaloid compounds, as well as terpenoids, amino acid and fatty acid derivates (19–21). The presence and concentration of these compounds may vary according to the diet and nutritional status of the insect in question, therefore constituting a relevant criteria when assessing their value as a dietary source (22, 23). From all entomochemicals, the best studied are the phenolics and their association with several biological activities.

### 1.2. Phenolic compounds in potentially edible insects

**FIGURE 1:**
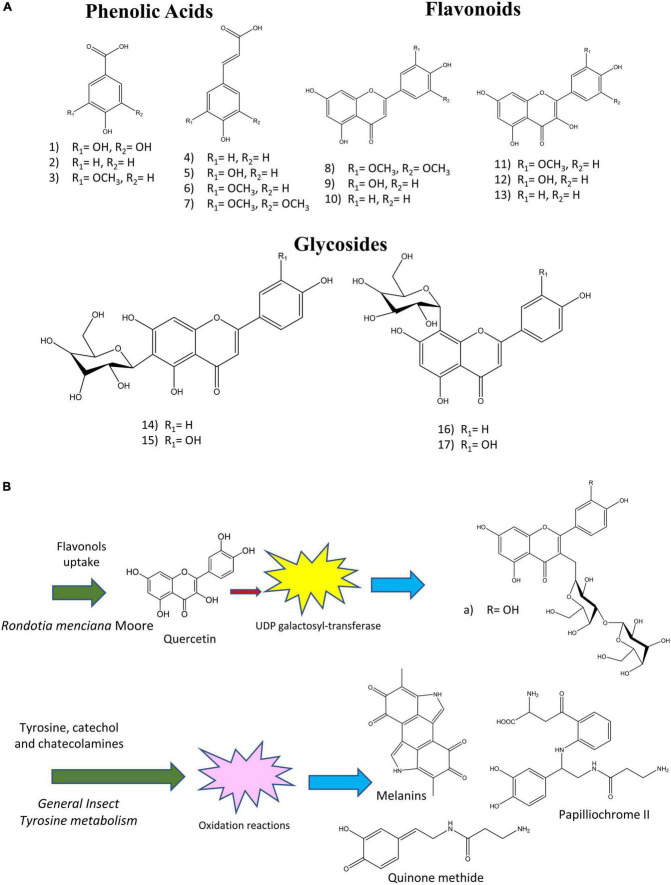
**(A)** Common phenolic compounds reported in edible insects. (1) gallic acid, (2) 4-hydroxybenzoic acid, (3) syringic acid, (4) p-coumaric acid, (5) caffeic acid, (6) ferulic acid, (7) sinapic acid, (8) tricin, (9) luteolin, (10) apigenin, (11) isorhamnetin, (12) quercetin, (13) kaempferol, (14) isovitexin, (15) iso-orientin, (16), vitexin, (17) orientin (1). **(B)** Examples of reported processing of phenolic compounds absorbed by insects with its derivatives. (a) Quercetin 3-O-β-D-galactopyranosyl-(1→3)-β-D-galactopyranoside (28–31).

Phenolic compounds are one of the more diverse and important groups with biological activity in nature; so much so, that they are currently considered as essential ingredients in food preparation, mainly because of their antimicrobial and antioxidant capacity. These phenolic compounds are characterized by their basic structure, which consists of a benzene ring bound to at least one hydroxyl radical. This group includes compounds such as phenolic acids, flavonoids, and tannins (24, 25). The presence of these compounds in edible insects is therefore highly relevant and has become the subject of research concerning their role as a source of nutrition, especially because these compounds can also provide color and taste to the prepared meals, adding further value and usefulness from the perspective of the food industry (26, 27) (Figure 1A).

Perhaps the association, between insects and bioactive phenolic compounds, was first established during their use as healing instruments throughout history in different cultures, with the prime example being Chinese traditional medicine, in which some insect species have been used as ancestral remedies in the treatment of diverse ailments and whose effectiveness has been later confirmed by modern medicine (32, 33). For example, Liubao tea is prepared by brewing *Hydrillodes morosa* Butler, *Nodaria niphona* Butler, *Aglossa dimidiate* Haworth, *Herculia glaucinalis* Linnaeus, and *Fujimacia bicoloralis* Leech. This infusion is commonly used to eliminate toxins, as a digestive, and to improve the overall health if the digestive tract. Previous reports suggest a clear effect over the expression of oxidative stress enzymes; further, *in vivo* studies have shown a protective effect on the gastric mucosa of mice (34). Another example is the case of *Holotrichia parallela* Motschulsky, this beetle usually invades crops of soy, sugar, and peanuts, among others, and it is mostly consumed throughout China and south-eastern Asia. Besides their nutritional value and rich content of antioxidant compounds, it has been traditionally used in the treatment of gout and mild infections (33, 35, 36). The exploration of phenolic compounds in edible insects has also led to the discovery of novel molecules with antioxidative and cytotoxic effects, the latter of which can affect cancer cells. In this regard, *Blaps rynchopetera* Fairmaire, a beetle in the family Tenebrionidae, has been used in Chinese traditional medicine in the treatment of cough, gastritis, and some types of cancer (37, 38). Ethanolic extractions from this species have rendered five rynchopeterines (A–E), in addition to other phenolic compounds associated with antioxidant activity, such as protocatechuic acid, 3,4-dihydroxyphenylacetic acid, 3,4-dihydroxybenzaldehyde, and 3,4-dihydroxyphenylacetaldehyde, among others (37). Recently, some reports have shown that the extracts obtained from *B. rynchopetera* have an immunomodulating effect; therefore, it has attracted greater interest in the fields of medicine and nutrition (39).

Other studies indicate that the presence of bioactive compounds in insects is directly correlated with their diet; however, it must be mentioned that these molecules are often modified within the insect itself (40). Further, it was discovered that some of these bioactive compounds are synthesized *de novo* through endogenous processes, among which are sclerotization and melanization, both of which include amino acid derivates and phenolic compounds (41–43) (Figure 1B). Therefore, the study of phenolic compounds in edible insects, i.e., their bioactive characteristics and stability during processing, is crucial to guarantee their nutritional value (Table 1). In this regard, previous studies have included insects of the orders Coleoptera, Hymenoptera, Isoptera, Diptera, Hemiptera, and Orthoptera.

**TABLE 1:** Phenolic and antioxidative compounds found in insects with potential application in food.

Insect species	Bioactive properties	Potential application	Type of compound	References
*Gryllodes sigillatus* Walker (Orthoptera)	Antioxidant	Baking goods	Various phenolic compounds	(27)
*Locusta migratoria* Linnaeus (Orthoptera)	Antioxidant	Energy bar	Various phenolic compounds, flavonoids and tannins	(44)
*Pterophylla beltrani* Bolívar and Bolívar (Orthoptera)	Antioxidant	Alcoholic beverages, tortillas	Various phenolic compounds	(26, 45)
*Ruspolia differens* Serville (Orthoptera)	Antioxidant	Cookies	Flavonoids	(46)
*Schistocerca piceifrons* Walker (Orthoptera)	Antioxidant	Alcoholic beverages	Various phenolic compounds	(47)
*Schistocerca gregaria* Foskal (Orthoptera)	Antioxidant	Cookies	Flavonoids	(48)
*Eulepida Mashona* Arrow (Coleoptera)	Antioxidant	Direct consumption	Flavonoids	(49)
*Rhynchophorus ferrugineus* Olivier (Coleoptera)	Antioxidant	Direct consumption, flour	Various phenolic compounds, flavonoids and tannins	(50)
*Tenebrio molitor* Linnaeus (Coleoptera)	Antioxidant	Baking goods, beverages, tortillas, direct consumption, flour	Various phenolic compounds	(27, 47, 50, 51)
*Zophobas morio* Fabricius (Coleoptera)	Antioxidant	Direct consumption, flour	Various phenolic compounds, flavonoids and tannins	(50)
*Antheraea pernyi* Guérin-Méneville (Lepidoptera)	Antioxidant	Direct consumption	Flavonoids	(52)
*Bombyx mori* Linnaeus (Lepidoptera)	Antioxidant	Direct consumption, flour	Various phenolic compounds	(53)
*Gonimbrasia belina* Westwood (Lepidoptera)	Antioxidant	Flour	Indetermined	(54)
*Macrotermes subhylanus* Rambur (Isoptera)	Antioxidant	Flour	Indetermined	(54)
*Odontotermes* sp. Holmgren (Isoptera)	Antioxidant	Direct consumption	Various phenolic compounds and flavonoids	(55)
*Encosternum delegorguei* Spinola (Hemiptera)	Antioxidant, antimicrobial	Direct consumption	Various phenolic compounds, flavonoids and tannins	(49)
*Oecophylla smaragdina* Fabricius (Hymenoptera)	Antioxidant	Direct consumption	Various phenolic compounds and flavonoids	(55)
*Hermetia illucens* Linnaeus (Diptera)	Antioxidant	Flour	Indetermined	(54)

### 1.3. Phenolic and antioxidative compounds in food

Concerning the inclusion and wide acceptance of insects in everyday diet, several proposals have been made showing the permanence and improved bioactive properties of food prototypes. For instance, the preparation of corn tortillas with the addition of powdered *Tenebrio molitor* Linnaeus specimens, which increased around 2% protein content, including essential amino acids (51). On the other hand, the incorporation of powdered *Pterophylla beltrani* Bolívar and Bolívar specimens in corn tortillas, at different ratios, increased the quantity of phenolic and antioxidative compounds, thus showing their potential benefit in everyday diet. Moreover, this also demonstrates the thermostability (∼100–115°C) of said compounds and their capacity to mix safely with the corn flour, from which they can be easily extracted if so desired. The addition of powdered insects does not affect the typical characteristics of corn tortillas; however, using concentrations close to 10% results in a darker color and their overall integrity is rather brittle (26).

Bakery products incorporating insect species in their preparation, such as muffins and cookies, has motivated their consumption as a nutritional source, usually because of their high protein content. Although the potential benefits of antioxidant compounds should not be neglected. The species *Gonimbrasia zambesina* Walker has been considered as a serious candidate in the preparation of enriched muffins because the addition of larvae significantly increases the protein content, among other nutritional parameters, up to 20%. However, the highest concentration that can be used is only of 10%, after which the product has poor consumer acceptance and shelf life (56, 57).

Further, the implementation of insects as baking ingredients should be critically considered due to their potential health hazards; therefore, the specimens should meet basic sanitary requirements to make them microbiologically safe (56). It must be considered that, although the inclusion of 10% insect powder (*T. molitor* and *Gryllodes sigillatus* Walker) in muffins and cookies results in a higher nutritional value, the quality of the resulting product is rather low concerning its taste (27, 58). This does not mean that the content of phenolic and antioxidative compounds is non-significant when used at a lower ratio; on the contrary, these molecules are preserved throughout the baking process, thus demonstrating their availability and bioactive potential as in the case of corn tortillas prepared with *P. beltrani*, which are processed at lower temperatures (26, 27). Interestingly, the cookies prepared with 10% powdered *T. molitor* and *G. sigillatus* shows a 1 mM Trolox equivalent antioxidant capacity when scavenging 2,2-diphenylpicrylhydrazyl (DPPH) and 0.3 mM for 2,2’-azino-bis(3-ethylbenzothiazoline-6-sulfonic acid) (ABTS) (27). This suggests that the antioxidant compounds found in these insects are mostly thermostable, providing versatility in the preparation of food and enhancing their integration potential in the human diet.

The use of insects as cooking ingredients can also be extended to beverages, which is in fact a common practice in several regions of the planet. In some places, this custom follows traditional practices and thus have a direct influence on their consumption and associated benefits. A clear example of this would be the preparation and consumption of mezcal, a distillated alcoholic beverage based on the fermentation of agave plants (59). In Mexico, agave worms (*Comadia redtenbacheri* Hammerschmidt) are typically consumed along with traditional alcoholic beverages, such as pulque, tequila, and mezcal. These worms, however, are not masticated but submerged in the alcohol, providing additional taste to the drink, and swallowed whole (60, 61). In this regard, the enrichment of alcoholic beverages such as rum, vodka, tequila, and mezcal with insects such as the Central American locust (*Schistocerca piceifrons* Walker), *T. molitor*, and *P. beltrani*, significantly increases the content of phenolic and antioxidative compounds that are notably stable, even after long-term storage at room temperature (45, 47). The consumption of insects in the form of infusions has been previously mentioned (30), thus hinting at their potential use in aqueous or ethanolic beverages to promote entomophagy in the common market. All of this is possible because both phenolic and antioxidative compounds can be easily extracted and integrated into most beverages, in addition to their protein content, which can be implemented in the preparation of baked goods.

## 2. Conclusion

The entomochemicals of phenolic nature derived from edible insects portray a unique opportunity to supply and improve high quality food, not as substitutes, but as an alternative to diversify and complement an adequate and functional diet. Currently, there is a trend on development foods supplemented with insects and is important to know all potential benefits that this implies, not only nutrients, also de nutraceuticals. As a bonus, the antioxidant activity associated with entomochemicals is rather stable throughout its processing for later consumption, thus opening the field of exploration, creativity, and innovation in the development of various foods, keeping insects as an effective cost-benefit option for both the producer and the consumer. Although entomophagy is an ancestral practice in many cultures around the world, its modern use still requires careful research prior to its widespread implementation to ensure its innocuity and nutritional value, thus diminishing the risk of allergic reactions or other health threatening side effects. Moreover, it requires strict quality control and sustainable strategies to establish a clear production and management method for these insects as a natural resource.

## Author contributions

JT-C wrote the initial draft and revised the manuscript. FO-S critically revised the manuscript. Both authors read and approved the manuscript.
